# Comparing the Denver criteria sets for blunt trauma: a retrospective study of cases in Edmonton, Alberta

**DOI:** 10.1259/bjr.20221116

**Published:** 2023-07-29

**Authors:** Mitchell J. Wagner, Imaan Hussein, Gavin Low, Karim Bahadurali Samji

**Affiliations:** 1 Faculty of Medicine and Dentistry, University of Alberta, Alberta, Canada; 2 Department of Diagnostic Imaging, University of Alberta, Alberta, Canada

## Abstract

**Objective:**

To determine whether a more conservative Denver criterion set could reduce unnecessary CT angiography (CTA) studies when screening for blunt cerebrovascular injury (BCVI) following blunt trauma.

**Methods:**

Following ethics approval, a retrospective chart review of 447 consecutive patients undergoing emergency CTA at two large teaching hospitals was conducted to determine the presence of risk factors for each Denver criterion set. Imaging studies of adults conducted between January 2016 and June 2020 containing sufficient clinical information for accurate classification were included in the study. Specificity, sensitivity, and predictive values were calculated. A two-sided Fisher exact test was used to evaluate the association between each iteration of the Denver criteria and the presence of BCVI.

**Results:**

The specificities of the Original, Modified, and Expanded Denver criteria were 43.58%, 34.32%, and 24.85%, respectively. Positive-predictive values (PPV) followed a different trend, with respective values of 2.77%, 3.06%, and 2.78%. Sensitivity and negative-predictive values (NPV) were found to be 100% for each criterion set. Being positive for a criterion set, and the presence of BCVI, was statistically significant for the original Denver criteria (*p* = 0.021, *n* = 443), but not the modified (*p* = 0.100, *n* = 345) or expanded Denver criteria (*p* = 0.202, *n* = 333).

**Conclusion:**

Use of the modified and expanded Denver criteria leads to the overuse of cerebrovascular imaging on patients suffering blunt force trauma.

**Advances in knowledge:**

The original Denver criteria may more appropriately identify subjects for further evaluation with CTA than the current standard, while retaining diagnostic efficacy for BCVI.

## Introduction

Blunt cerebrovascular injury (BCVI) is a term that describes blunt trauma to the carotid or vertebral arteries and is thought to be caused by hyperflexion/extension of the neck.^1–4^ BCVI was initially perceived to be infrequent in blunt trauma populations with an incidence as low as 0.08%, but with extensive screening protocols, its incidence has risen to up to 2.99%.^3,5–7^ BCVI can be directly attributed to mortality of patients suffering blunt trauma as demonstrated by Berne et al, reporting that the overall mortality rate of patients with BCVI was 59% in their study population, with 80% of those deaths directly attributed to BCVI.^4^ A primary contributor to the mortality caused by BCVI is stroke, with rates varying from 10 to 13% in patient populations suffering from BCVI.^3,7,8^

BCVI-related complications have been impacted by the adoption of screening protocols. In the aforementioned study by Berne et al, it was noticed that a longer time to diagnosis of BCVI correlated with greater morbidity,^4^ emphasizing the need for prompt BCVI screening. Additionally, adoption of screening protocols has been correlated with a greater incidence of BCVI, a drop in neurological insults, and a significant drop in stroke and mortality rates associated with BCVI.^3,5,8^ Therefore, screening protocols that can help identify asymptomatic individuals with BCVI before complications occur are of great utility. The Denver screening protocol aims to screen for BCVI, recommending that patients suffering blunt force trauma to the head and neck be screened using CT angiography (CTA) if positive for one or more screening criteria.^3^ The Western and Eastern Trauma Associations in 2009 and 2010, respectively, have recommended the modified Denver criteria,^9,10^ but have both recognized that some institutions adopt more liberal screening practices to capture individuals lacking screening criteria. The expanded Denver criteria (Table 1) are recommended by Brommeland et al as best practice.^1,11^ Brommeland and colleagues concede that while the expanded Denver criteria can contribute to identifying the 20–30% of patients without identifiable criteria in earlier iterations of the Denver protocol,^7,11^ the risks of over imaging patients and delivering unnecessary radiation doses are a concern.^1^

**Table 1.** Iterations of the Denver screening protocol. Blunt trauma victims positive for one or more criteria are recommended to have CT angiography

Denver Criteria	Modified Denver Criteria	Expanded Denver Criteria
Le Fort II or III	Le Fort II or III	Le Fort II or III
Complex skull fracture/basilar skull fracture/occipital condyle fracture	Complex skull fracture/basilar skull fracture/occipital condyle fracture	Complex skull fracture/basilar skull fracture/occipital condyle fracture
Severe traumatic brain injury (TBI) with GCS < 6	Severe traumatic brain injury (TBI) with GCS < 6	Severe traumatic brain injury (TBI) with GCS < 6
Cervical spine fracture, subluxation or ligamentous injury at any level	Cervical spine fracture, subluxation or ligamentous injury at any level	Cervical spine fracture, subluxation or ligamentous injury at any level
Near hanging with anoxic brain injury	Near hanging with anoxic brain injury	Near hanging with anoxic brain injury
	Seat belt abrasion with significant swelling, pain, or altered mental status	Seat belt abrasion with significant swelling, pain, or altered mental status
		TBI with thoracic injury
		Scalp degloving
		Thoracic vascular injury
		Blunt cardiac rupture
		Upper rib fracture (ribs 1–6)

Optimization of screening criteria to maximize sensitivity of BCVI screening while minimizing unnecessary radiation exposure and use of clinical resources is yet to be elucidated.^1,3,6^ Previous studies have sought to optimize the screening protocols for sensitivity of BCVI; however, there seems to be a lack of studies comparing the iterations of the Denver criteria.^1^ Therefore, the objective of this study was to compare the iterations of the Denver criteria protocols side-by side-to compare efficacy for BCVI screening. Furthermore, we investigated whether the expanded Denver criterion leads to overimaging of blunt trauma populations in comparison with earlier iterations and evaluated the association of individual expanded Denver criteria with BCVI. It is hypothesized that, if the expanded Denver criteria’s broader criteria set (Table 1) leads to overimaging, one would expect a decreased specificity for BCVI without a significant increase in sensitivity in comparison with the original and modified criteria.

## Methods

A retrospective review of radiology reports of subjects over the age of 18 undergoing emergency computed tomography angiography (CTA) between January 1, 2016 and June 30, 2020 was conducted by authors MW and IH. Power analysis was carried out to anticipate the minimum sample size for the study. Data were obtained from two large quaternary teaching hospitals in Canada, yielding a total of 447 cases. Imagers were all reviewed by a team of fellowship-trained neuroradiologists. Subjects suffering from penetrating trauma were excluded from the study. Subjects were evaluated using each of the Denver criteria iterations (Table 1), allowing calculation of the specificity, sensitivity, positive predictive value (PPV), and negative predictive value (NPV) for each Denver criterion set. All CTs during the study period were performed with a 64-slice Toshiba Aquilion 64 scanner, with identical imaging protocols. CTA imaging is performed with a slice thickness/interval of 2 x 2 mm. Axial, coronal, and sagittal images are reconstructed at 3 x 1 mm slice thickness/interval. During the exam, 100 cc of contrast is administered at 5 cc/s. Cases lacking insufficient clinical or diagnostic information to determine the presence of risk factors from each of the Denver criteria iterations were excluded from the study (supp figure 1). We excluded four cases from our study population before applying the original Denver criteria, 102 before applying the modified Denver criteria, and 114 cases before applying the expanded Denver criteria. A two-sided Fisher’s exact test and a two-sided Pearson chi-squared test were used to determine the association between positivity for one or more Denver criteria risk factors and the presence of BCVI following blunt trauma. Additionally, effect sizes (phi values), Cramer’s V test, and likelihood ratios were also calculated for each of the Denver criteria sets in relation to BCVI. A two-sided Fisher’s exact test was also used to evaluate the association with BCVI of each individual criterion.

Supplementary Figure 1.

## Results

**Figure 1.**
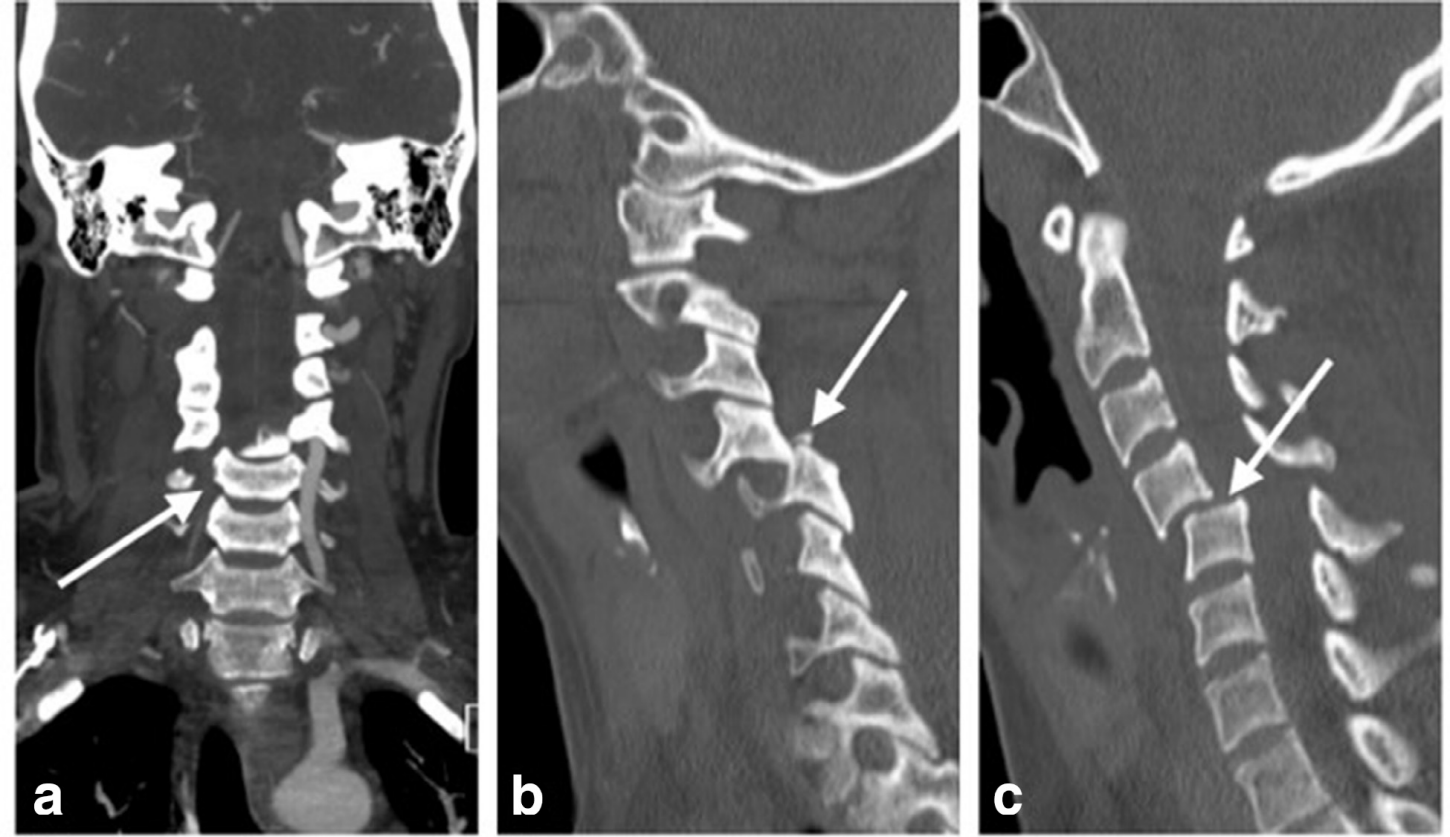
31-year-old female who sustained a blow to the head. **a** coronal CTA image demonstrating a ringt vertebral artery dissection (arrow). This extended form the level of the C3/4 interspace to the C6 vertebral body. **b**. Sagittal reconstruction with done displaced anterior to the superior facet of the C5 vertebral body,in keeping with a jumped/locked facet joint. A mildly displaced tiny fracture fragment is also demonstrated at the tip of the superior facet of the C5 vertebral body (arrow). **c**. Sagittal reconstruction with bone windows at the level of the midline demonstrates grade 2 anterolisthesis of C4 over C5 (arrow).

**Figure 2.**
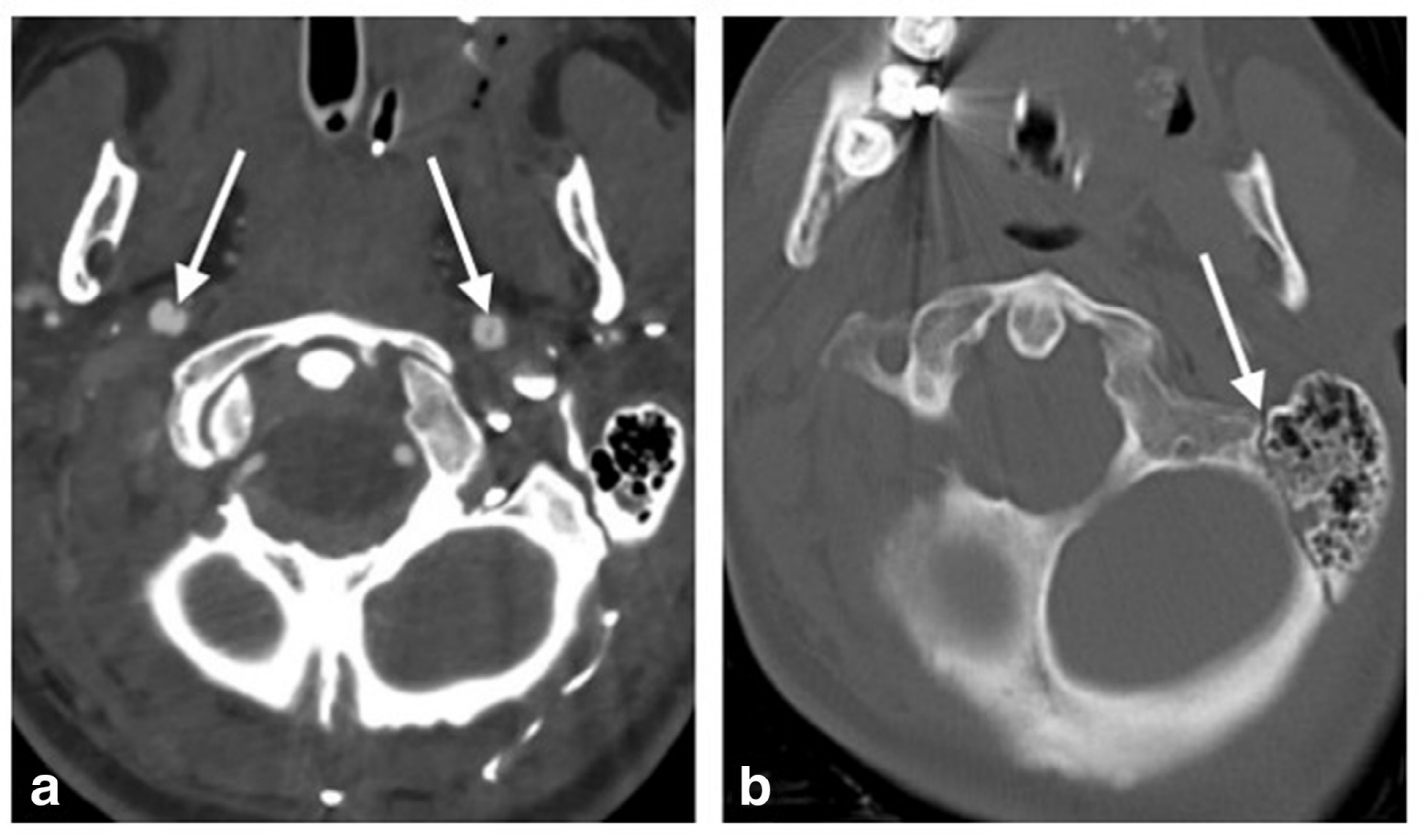
50-year-old female involved in accident whilst riding her motorcycle. **a**. Axial CTA image demonstrating bilateral internal carotid artery dissections at the C1 level (arrows). **b**. Axial image in bone windows demonstrate a left base of skull fracture involving the left temporal bone (arrow).

Our study included *n* = 447 total cases. From our data, *n* = 7 subjects were found to suffer from BCVI for an incidence rate of 1.56%. The original, modified, and expanded Denver criterion sets were applied to *n* = 443 (4 cases excluded), *n* = 345 (102 cases excluded), and *n* = 333 (114 cases excluded) of the total cases, respectively, after excluding patients lacking scans that would allow evaluation with a Denver criterion set (Supplementary Material 1). A two-sided Fisher’s test was used to analyze the association between BCVI and positivity for one or more Denver criteria in each set. The only statistically significant correlation (*p* < 0.05) was found for the original Denver criteria (*p* = 0.021). The modified and expanded criterion sets were not statistically significant, with the modified set having a lower *p*-value (*p* = 0.100) than the expanded set (*p* = 0.202) (Table 2). These results were also evaluated using a chi-squared test, likelihood ratios, and Cramer’s V test, yielding the same *p*-value (Table 2). The original, modified, and expanded Denver criteria sets all obtained sensitivities of 100% within our dataset. Specificity followed a downward trend, calculated to be 43.58%, 34.32%, and 24.85% for the respective criterion sets. The calculated NPV for each criterion set was 100%. The calculated PPV is 2.77%, 3.06%, and 2.78% for the respective criterion sets (Table 2). Within the seven cases that were positive for BCVI, the only criteria that were present in these individuals was complex skull fractures (2/7, 29%); cervical spine fracture, ligamentous injury, or subluxation (4/7, 57%); thoracic vascular injury (1/7, 14%); and upper rib fractures (2/7, 29%) (Table 3). Examples of positive BCVI are shown in Figures 1 and 2.

Supplementary Material 1.

**Table 2.** Sample sizes and calculated statistics for application of each Denver criterion set

Statistic / Criterion set	Original Denver	Modified Denver	Expanded Denver
Sample size (n)	443	345	333
Sensitivity	100%	100%	100%
Specificity	43.58%	34.32%	24.85%
Positive Predictive Value (PPV)	2.77%	3.06%	2.78%
Negative Predictive Value (NPV)	100%	100%	100%
Fisher’s exact test (*p*-value)	0.021	0.100	0.202
Phi value (effect size)^a^	0.11	N/A	N/A

a Phi values (effect sizes) not reported for statistically insignificant associations.

**Table 3.** Association of individual Denver criteria with BCVI

Criterion	Proportion of BCVI cases positive for criterion	Fisher’s exact test (*p*-value)
Le Fort II or III	0/7	1.0
Complex skull fracture/basilar skull fracture/occipital condyle fracture	2/7 (29%)	0.321
Severe traumatic brain injury (TBI) with GCS < 6	0/7	1.0
Cervical spine fracture, subluxation or ligamentous injury at any level	4/7 (57%)	0.433
Near hanging with anoxic brain injury	0/7	1.0
Seat belt abrasion with significant swelling, pain or altered mental status	0/7	1.0
Thoracic vascular injury	1/7 (14%)	0.061
Upper rib fracture (ribs 1–6)	2/7 (29%)	0.681

## Discussion

The purpose of the study was to compare the diagnostic performance of each iteration of the Denver criteria at screening for BCVI. It was hypothesized that the broader, expanded Denver criteria would result in lower specificities without significantly affecting sensitivity, leading to over imaging. This hypothesis was confirmed in our statistical analysis. As the Denver criterion set broadened, the specificity dropped correspondingly. Sensitivity remained constant amongst each of the three Denver criterion sets. Additionally, statistical analysis showed a significant correlation between application of the original Denver criteria and detection of BCVI, whereas application of the modified or expanded criterion sets were not significantly associated. Although the high sensitivity demonstrated in our data set is a desirable characteristic, it seems application of the modified or expanded criterion sets did not add to the diagnostic yield of the Denver protocol. Application of the original Denver criteria yielded higher specificity and was significantly associated with BCVI.

Throughout the history of the Denver criteria’s development and application, a balance has been sought between the sensitivity for detecting BCVI, afforded by the broadness of the criteria set, with its specificity, which reduces screening cost.^6^ The ‘holy grail’ of the field would be to know which criterions within a particular Denver criteria set specifically contribute to a lack of specificity, yet contribute to sensitivity. Our study shows some findings in this regard. By comparing the specific criteria that make up these sets, this would suggest that the extra criteria within the modified and expanded Denver criteria do not contribute to the diagnostic yield of the Denver criteria for BCVI screening. This would include seatbelt abrasion with altered mental status, upper rib fractures, traumatic brain injury (TBI) with thoracic injury, scalp degloving, thoracic vascular injury, or blunt cardiac rupture. Of these ‘extra’ criteria to the original Denver criteria set, upper rib fractures specifically seemed to be a common appearance in our cohort which may have reduced the expanded Denver criteria’s specificity for BCVI. Furthermore, this criterion was not significantly associated with BCVI via our statistical testing (Table 3), although it is acknowledged that we have a small sample size. It stands to reason that rib fractures, although they may be common in patients who have experienced trauma, are not necessarily in line with the mechanism of injury that causes BCVI^3^ and thus may not be predictive in screening protocols. This is reinforced by a large cohort study of BCVI conducted in 2021 focusing on TBI. They note that cervical spine fractures, mandible fracture, basilar skull fractures, and neck contusion were most associated with BCVI, rather than upper rib fractures.^12^

Our data concur with numbers previously reported in the literature. An incidence rate of 1.56% obtained from our patient population is within the published range of BCVI incidence, thought to be 1.20 to 2.99%.^3^ In a study by Beliaev et al, which also aimed to evaluate the efficacy of Denver criterion sets’ ability to screen for BCVI, found that evaluation of patients with the Denver protocol yielded a sensitivity of 97%, specificity of 42%, a PPV of 4.43%, and an NPV of 99.8% for BCVI.^6^ These numbers are similar to our findings when applying the original Denver protocol. Our study additionally found no significant association between any individual criteria and BCVI (Table 3). These results are consistent with a study done by Tso et al, which also found a lack of statistical association between any single Denver criterion and BCVI. They also note that only 7% of their population who displayed risk factors were diagnosed with BCVI.^13^ This result, with ours, reflects the heterogeneity of risk factors in patients suffering from BCVI and a need to further optimize screening protocols. It may be that the existence of two or more risk factors in tandem within the criteria set better predict a patient’s risk of developing BCVI. This is an area which appears under explored within the literature and could be a further direction.

The NPV of 100% in our study would seem to indicate that a negative result screening with any Denver criterion set excludes BCVI every time. Similarly, our obtained sensitivities were 100%, implying that screening via CTA can capture BCVI 100% of the time. Whilst these values are expected to be high, they may not be truly representative, given our relatively small sample size. In comparison, Beliaev and colleagues report values of 97% sensitivity and 89% negative predictive value, looking at a significantly larger pool of 18 patients with BCVI.^6^

The necessity of appropriate screening protocols has only become more evident with the development of an increased understanding of BCVI.^14,15^ While these injuries were initially rarely characterized, diagnosis has only become more common over time.^3,5–7^ Since adequate treatment in BCVI patients has shown promise in preventing stroke, screening programs are of utmost importance. Nevertheless, the significance of such screening protocols is still uncertain.^13^ Our retrospective analysis of patients undergoing emergency CTA imaging provided crucial information about the application of specific Denver criterion. Through this study, we determined that there is no significant difference in the ability of any of the individual screening criteria in distinguishing between patients with or without BCVI.

This study was not without limitations. Our research is not only subject to the associated biases with retrospective studies, but also a lack of follow up with patients to ensure that BCVI did not develop later or that false negatives were not missed. Although incidence of BCVI was within expected ranges purported by the literature and our study encompassed multiple trauma centers, a small number of cases with BCVI in the study cohort poses a challenge for generalizability. In order to apply the Denver criteria rigorously to our patient population, some cases needed to be excluded based on a lack of clinical data needed to discern positivity for a given Denver criterion. Thus, a limitation was placed on which blunt trauma cases could be included in the study.

## Conclusion

In conclusion, our results suggest that the application of the modified and expanded Denver criteria contributes to over imaging of patients with CTA. We also establish that application of the expanded or the modified Denver criteria did not meaningfully improve diagnostic yield in comparison to the original Denver criteria. Further research should continue to explore new combinations of criterions within the Denver criteria sets to provide an optimal approach to BCVI screening that maximizes diagnostic yield and minimizes over triage.
