# Supplementary figures and images for: Comparing the Denver criteria sets for blunt trauma: a retrospective study of cases in Edmonton, Alberta
Source: Br J Radiol. 2023 Jul 29;96(1148):20221116. doi: 10.1259/bjr.20221116 (PMC10392660; doi:10.1259/bjr.20221116)

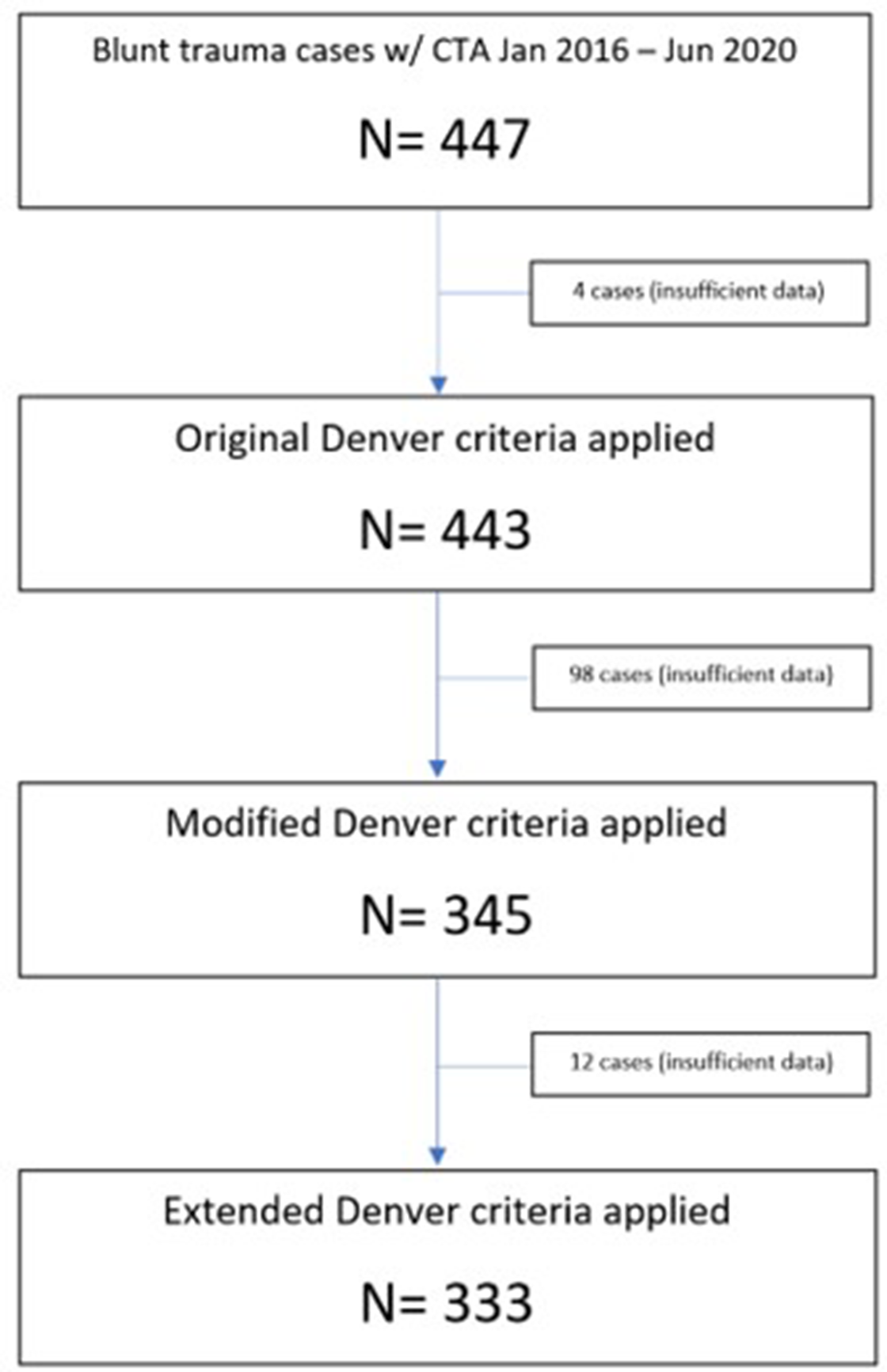

Supplement: Supplementary Figure 1. [file bjr.20221116.suppl-01.tif]
