# Supplementary material for: Comparing the Denver criteria sets for blunt trauma: a retrospective study of cases in Edmonton, Alberta
Source: Br J Radiol. 2023 Jul 29;96(1148):20221116. doi: 10.1259/bjr.20221116 (PMC10392660; doi:10.1259/bjr.20221116)
Supplement: Supplementary Material 1. [file bjr.20221116.suppl-02.docx]

https://www.birpublications.org/page/preparing/bjr#ref

<https://www.equator-network.org/wp-content/uploads/2015/10/STROBE_checklist_v4_cohort.pdf>

| **Section/topic** | **Item number** | **Recommendation** | **Status** |
| --- | --- | --- | --- |
| Title and abstract | 1 | Indicate the study’s design with a commonly used term in the title or the abstract  Provide in the abstract an informative and balanced summary of what was done and what was found | Done |
| Introduction |  |  |  |
| Background/rationale | 2 | Explain the scientific background and rationale for the investigation being reported | Done |
| Objectives | 3 | State specific objectives, including any prespecified hypotheses | Done |
| Methods |  |  |  |
| Study design | 4 | Present key elements of study design early in the manuscript | Done |
| Setting | 5 | Describe the setting, locations, and relevant dates, including periods of recruitment, exposure, follow-up, and data collection | Done |
| Participants | 6 | Cohort study - give the eligibility criteria, and the sources and methods of selection of participants; describe methods of follow-up  Case-control study - give the eligibility criteria, and the sources and methods of case ascertainment and control selection; give the rationale for the choice of cases and controls  Cross-sectional study - give the eligibility criteria, and the sources and methods of selection of participants  Cohort study - for matched studies, give matching criteria and number of exposed and unexposed Case-control study - for matched studies, give matching criteria and the number of controls per case | Done |
| Variables | 7 | Clearly define all outcomes, exposures, predictors, potential confounders, and effect modifiers; give diagnostic criteria, if applicable | Done |
| Data sources/measurement | 8* | For each variable of interest, give sources of data and details of methods of assessment (measurement); describe comparability of assessment methods if there is more than one group | Done |
| Bias | 9 | Describe any efforts to address potential sources of bias | Done |
| Study size | 10 | Explain how the study size was arrived at | Done |
| Quantitative variables | 11 | Explain how quantitative variables were handled in the analyses; if applicable, describe which groupings were chosen and why |  |
| Statistical methods | 12 | Describe all statistical methods, including those used to control for confounding  Describe any methods used to examine subgroups and interactions  Explain how missing data were addressed  Cohort study - if applicable, explain how loss to follow-up was addressed  Case-control study - if applicable, explain how matching of cases and controls was addressed  Cross-sectional study - if applicable, describe analytical methods taking account of sampling strategy  Describe any sensitivity analyses | Done |
| Results |  |  |  |
| Participants | 13* | Report numbers of individuals at each stage of study - e.g., numbers potentially eligible, examined for eligibility, confirmed eligible, included in the study, completing follow-up, and analyzed  Give reasons for nonparticipation at each stage Consider use of a flow diagram | Done |
| Descriptive data | 14* | Give characteristics of study participants (e.g., demographic, clinical, social) and information on exposures and potential confounders  Indicate number of participants with missing data for each variable of interest Cohort study - summarize follow-up time (e.g., average and total amount) | Done |
| Outcome data | 15* | Cohort study - report numbers of outcome events or summary measures over time  Case-control study - report numbers in each exposure category, or summary measures of exposure  Cross-sectional study - report numbers of outcome events or summary measures | Done |
| Main results | 16 | Give unadjusted estimates and, if applicable, confounder-adjusted estimates and their precision (e.g., 95% confidence interval); make clear which confounders were adjusted for and why they were included Report category boundaries when continuous variables were categorized  If relevant, consider translating estimates of relative risk into absolute risk for a meaningful time period | Done |
| Other analyses | 17 | Report other analyses done - e.g., analyses of subgroups and interactions, and sensitivity analyses | Done |
| Discussion |  |  |  |
| Key results | 18 | Summarize key results with reference to study objectives | Done |
| Limitations | 19 | Discuss limitations of the study, taking into account sources of potential bias or imprecision; discuss both direction and magnitude of any potential bias | Done |
| Interpretation | 20 | Give a cautious overall interpretation of results considering objectives, limitations, multiplicity of analyses, results from similar studies, and other relevant evidence | Done |
| Generalizability | 21 | Discuss the generalizability (external validity) of the study results | Done |
| Other information |  |  |  |
| Funding | 22 | Give the source of funding and the role of the funders for the present study and, if applicable, for the original study on which the present article is based | Done |
